# Building integrated, adaptive and responsive healthcare systems – lessons from paramedicine in Ontario, Canada

**DOI:** 10.1186/s12913-022-07856-z

**Published:** 2022-05-03

**Authors:** Amir Allana, Kerry Kuluski, Walter Tavares, Andrew D. Pinto

**Affiliations:** 1 Institute of Health Policy, Management and Evaluation, Dalla Lana School of Public Health, University of Toronto, ON Toronto, Canada; 2 McNally Project for Paramedicine Research, Toronto, Canada; 3 Upstream Lab, MAP/Centre for Urban Health Solutions, Li Ka Shing Knowledge Institute, Unity Health Toronto, Toronto, Canada; 4 Institute for Better Health, Trillium Health Partners, Mississauga, Canada; 5 The Wilson Centre and Temerty Faculty of Medicine, University of Toronto | University Health Network, Toronto, Canada; 6 York Region Paramedic Services, Community and Health Services Department, The Regional Municipality of York, Canada; 7 Department of Family and Community Medicine, Faculty of Medicine, University of Toronto, Toronto, Canada; 8 Department of Family and Community Medicine, St. Michael’s Hospital, Toronto, Canada

**Keywords:** Integrated care, Paramedic, Complexity, Adaptive, Responsive, Governance, Regulation, Qualitative, Health services

## Abstract

**Background:**

Being responsive and adaptive to local population needs is a key principle of integrated care, and traditional top-down approaches to health system governance are considered to be ineffective. There is need for more guidance on taking flexible, complexity-aware approaches to governance that foster integration and adaptability in the health system. Over the past two decades, paramedics in Ontario, Canada have been filling gaps in health and social services beyond their traditional mandate of emergency transport. Studying these grassroots, local programs can provide insight into how health systems can be more integrated, adaptive and responsive.

**Methods:**

Semi-structured interviews were conducted with people involved in new, integrated models of paramedic care in Ontario. Audio recordings of interviews were transcribed and coded inductively for participants’ experiences, including drivers, enablers and barriers to implementation. Thematic analysis was done to ascertain key concepts from across the dataset.

**Results:**

Twenty-six participants from across Ontario’s five administrative health regions participated in the study. Participants described a range of programs that included acute, urgent and preventative care driven by local relationship networks of paramedics, hospitals, primary care, social services and home care. Three themes were developed that represent participants’ experiences implementing these programs in the Ontario context. The first theme, *adapting and being nimble in tension with system structures*, related to distributed versus central control of programs, a desire to be nimble and skepticism towards prohibitive legal and regulatory systems. The second theme, *evolving and flexible professional role identity*, highlighted the value and challenges of a functionally flexible workforce and interest in new roles amongst the paramedic profession. The third theme, *unpredictable influences on program implementation*, identified events such as the COVID-19 pandemic and changing government priorities as accelerating, redirecting or inhibiting local program development.

**Conclusions:**

The findings of this study add to the discourse on governing health systems towards being more integrated, adaptive and responsive to population needs. Governance strategies include: supporting networks of local organizational relationships; considering the role of a functionally flexible health workforce; promoting a shared vision and framework for collaboration; and enabling distributed, local control and experimentation.

## Background

A system that is responsive to the health and social needs of local populations is a key goal of integrated care [1], but leaders and policy makers face challenges building systems that are adaptive and flexible [2]. ‘Integrated care’ can be defined as when networks of health and social services are coordinated to meet the needs of individual clients, and account for communities’ social, cultural, geographic and population health contexts [1, 3]. Given their focus on meeting diverse, changing needs in diverse contexts, integrated care systems rely on local providers to continually understand their target populations and adapt services accordingly [2]. Failure to do so leads to inappropriate and disconnected services, particularly between primary, acute and social care. This, coupled with poor health information flow, can result in negative client experiences, missed opportunities for disease prevention and costly inefficiencies [4, 5].

Health services researchers have suggested that challenges in implementing integrated care are partly due to a top-down, ‘command and control’ approach to managing health systems, rather than a flexible, ‘hands-off’ approach that embraces the messiness and uncertainty inherent in complex systems [2, 6]. For instance, research on network governance has identified how different decentralized configurations in organizational relationships, leadership structures and trust contribute to shared action in health systems [7]. More recently, the principles of complexity thinking have been discussed in terms of the impact of shared mental models held by health system actors on transformation efforts, and mechanisms to foster experimentation and adaptability in health systems [2, 6, 8]. Both network governance and complexity thinking allude to navigating tensions between centralized and distributed leadership, innovation and standardization, and the role of rules, policy and regulation. However, there continue to be limited examples and guidance on how to enact these principles in practice, stymying efforts to integrate care.

In Ontario – Canada’s most populous province – responsibilities for funding and administering health and social services are distributed amongst different levels of government (municipal, provincial and federal) and include publicly-funded, not-for-profit and private providers [9]. Providers are spread across Ontario’s vast geography that include urban, sub-urban, rural and remote communities. This complex distribution of legislation, funding, organizations and jurisdictions have been the backdrop for multiple health system reform efforts over the past two decades. In 2006, the Ontario government created Local Health Integration Networks (LHINs) to contract and coordinate community-based home care services in regional service areas. Despite the implementation of LHINs, silos continued to persist leading to gaps in services and inadequate care [2, 10]. In 2018, the government announced a dissolution of LHINs, to be replaced with a new system of integrated care which include the formation of Ontario Health Teams (OHTs) [11]. OHTs are meant to be geographically-based, self-governing networks of providers that include primary care, hospital and community-based services [11]. While the intention is for OHTs to identify and respond to local population needs, it is unclear what combinations of roles, providers and organizations might best enable them to do so, and a need for more guidance on governance approaches to facilitate collaboration in these integrated care provider networks [12].

Amongst a continuously changing health system landscape over the past two decades, there have been a proliferation of new services provided by paramedics in Ontario in response to local service needs [13]. Beyond their traditional role of emergency response and transport, paramedics have been providing a growing list of preventative, community-based and chronic disease care [13, 14]. These services are often nebulously termed ‘community paramedicine’ (CP) [14]. These diverse CP programs have been referred to as examples of integrated care [15], but face challenges with scope and definition, leading some to question whether they are simply ad-hoc “patches” to a broken system rather than “well-thought-out” improvements [﻿^16^ p. 691]. Beginning in 2014, the Ontario government started responding to CP by providing project grants and updating regulations [17, 18]. The paramedic community has also indicated an interest in better integrating with health and social care systems [19], but their inclusion in OHTs has so far been variable.

The grassroots emergence of new models of paramedic care – and the Ontario government’s response to them – provides a rich context to explore how health systems adapt to local needs and the role of paramedics in integrated care. Studying this can provide insight into how to systematically support integrated care efforts more broadly, and add to the literature on health system governance and transformation [6, 20, 21]. This study examined the experiences and perspectives of people involved with implementing ‘non-traditional’ models of paramedic practice in Ontario over the past two decades using the lens of integrated care. We set out to answer the following research questions: What have been the drivers, enablers and barriers to enacting new ‘integrated’ models of paramedic care in Ontario? What does this suggest for governing health systems towards being more integrated?

## Methods

### Overview

This study was rooted in a constructivist qualitative paradigm and borrowed from the field of phenomenology. It was constructivist because ‘meaning’ was constructed through conversations between the researcher and participants, and the results are an interpretation of the data. It was phenomenological in the sense that we looked to understand common patterns in peoples’ experiences. We purposively recruited participants who had been involved in implementing new models of paramedic care in Ontario, and asked them about their experiences implementing these programs. Data was collected via audio-recorded one-on-one semi-structured interviews. After transcription and de-identification, interview transcripts were coded inductively and analyzed in two ways: qualitative description and thematic analysis. Descriptive and thematic results were both reported. The study team’s expertise in integrated care, paramedicine and primary care influenced how results were interpreted. The study lead was a paramedic in the Ontario context; this insider perspective meant having shared language and trust with participants and access to professional networks for recruitment.

### Ethics

Ethics approval was obtained from the University of Toronto Research Ethics Board (protocol # 40127) prior to recruiting participants. All participants gave informed consent and data collection proceeded as per processes pre-approved by the ethics board.

### Recruitment and sampling

Purposive and snowball sampling strategies were used to identify and recruit potential participants [22], and geographic variation was maximized where possible. We started by compiling a list of program managers or coordinators of new models of paramedic care in Ontario using publicly-available reports [13]. The study lead (AA) contacted these individuals by email, directly or through professional networks, using a scripted invitation and an information package about the study. At the end of each interview, participants were asked to recommend other potential participants, and recruitment continued iteratively in this way. We tried, where possible, to recruit from diverse geographic regions across Ontario to capture perspectives from across the province. Data saturation [23] was monitored throughout data collection, with informal coding taking place after each interview and a memo of findings maintained in a working document. Three times during data collection, based on informal coding and memos, preliminary candidate themes were developed and discussed by the study team. Data collection continued until little new or repeat data were being produced, no new themes were being identified and the dataset was sufficiently detailed to describe each idea. In this way, we defined ‘saturation’ to have been reached when further data collection would produce diminishing returns to the richness and completeness of our analysis [24].

### Data collection

Consenting participants were invited to one-on-one, semi-structured interviews with the study lead (AA). The interview guide was structured such that it started with broad and open-ended questions, and then included some more specific prompts to be used by the interviewer as-needed. After reminding participants of the goals of the study, the interviewer asked them to identify what programs and models of care they had been involved with that they would like to speak about. They were asked to describe how and why these programs were created, their experiences in implementing them, and any enablers and barriers they encountered along the way. Additional prompts in the interview guide were consulted if needed to stimulate the conversation. All interviews were done remotely via Zoom videoconferencing (http://www.zoom.us); they were scheduled for 1 hour but allowed to end naturally. Audio recordings of the interviews were transcribed verbatim, reviewed for accuracy, deidentified and loaded into QSR NVivo 12 (http://www.qsrinternational.com/) for analysis.

### Analysis and reporting

Transcripts were coded openly and inductively, and analyzed in two stages: (a) qualitative description [25, 26] to report on drivers, enablers and barriers; and (b) thematic analysis [27] to distill core ideas from across the dataset. Transcripts were coded for words, phrases and meanings that represented elements of participants’ experiences while implementing new models of paramedic care. No pre-existing framework was imposed on the data and any experiences participants described were coded for, but we were also deliberately interpreting for drivers, enablers and barriers encountered during program implementation. An initial set of five transcripts were coded simultaneously by three members of the study team and a preliminary codebook was developed. The remainder of the coding was completed by AA and the entire study team met repeatedly to discuss transcripts, codes and interpretations. Similar codes were clustered together and categorized to report the drivers, enablers and barriers participants experienced. Following Braun and Clarke’s method for thematic analysis [27], all codes were aggregated into themes that cut across the data set. Themes were revised and reorganized iteratively by examining the data under them; the final themes and codes were checked for consistency, coherency and completeness. Hierarchy and relationships between the themes were determined; this was reflected by some themes being reported as sub-themes, and in the narrative of results. Representative quotes were chosen that exemplify themes and key concepts. In order to be concise when reporting results, representative quotes were truncated where possible; this is indicated by “[ …]” where applicable. Qualitative rigor was maintained by continuously re-examining data, independent reading of transcripts by members of the study team, transparent reporting of methods, and writing memos throughout data collection and analysis to continually reflect on decisions, researcher preconceptions and interpretations [28].

## Results

Over the course of recruitment, 30 people were invited, of which 26 responded and agreed to participate. Twenty-four interviews were conducted, lasting approximately 1 hour on average (one group interview with three participants, on participants’ request). A summary of participant characteristics is provided in Table 1. A slight majority of participants were male (54%), most had professional backgrounds as paramedics (65%) or physicians (19%), and all were in management or leadership positions. All five health regions in Ontario – as defined by the Ministry of Health – were represented in the sample; Ontario’s West region was over-represented (35%) while the North was under-represented (15%). In the sections below, a description of program types, drivers, enablers and barriers are provided first, followed by thematic analysis of key concepts from across the dataset.

**Table 1:** Interview participant demographics

	***n***	***%***
**Sex**		
Male	14	54%
Female	12	46%
**Geographic Region in Ontario**		
West	9	35%
Central & Toronto	7	27%
East	6	23%
North	4	15%
**Health Profession**		
Paramedic	17	65%
Physician	5	19%
Nursing, Occupational or Physical Therapist	2	8%
*Missing or not applicable*	2	8%
**Main Organizational Affiliation**		
Municipal or regional paramedic service	15	58%
Ontario Health or Local Health Integration Network	5	19%
Base hospital (regulatory body)	3	12%
Hospital	2	8%
Primary care organization	1	4%
	***Years***	
**Healthcare Experience**		
Mean	20.0	
Range	7 - 40	

### Description of programs, drivers, enablers and barriers

Participants described a range of programs and models of care, listed in Table 2. Programs provided both emergent and preventative care services in community settings, including unscheduled response to exacerbations in chronic symptoms, scheduled home visits, mobile drop-in clinics and vaccination campaigns. Target populations ranged from broad (e.g., anyone experiencing a mental health crisis) to specific (people enrolled in palliative care or chronic disease management programs). As seen in Table 2, target populations and types of services varied and were influenced by local context (e.g., rural and remote areas versus urban).

**Table 2:** Descriptions of new models of paramedic care in Ontario

Category or target population	Program characteristics and typical activities
Chronic disease management and aging at home	Paramedics supporting primary and home care teams – including physicians, nurses, social workers and personal support workers – to provide individualized preventative and emergent care to clients at home. Target populations include those with heart and lung disease, diabetes and dementia. Services include home wellness visits, blood and urine analysis, medication adjustment and symptom management.
Community outreach and harm reduction	Paramedics supporting mobile clinics, outreach and harm reduction programs in community housing, shelters and street settings. Health prevention, promotion and safe consumption services for range of populations: seniors; people on social assistance; people who are homeless or undocumented; people with substance use disorders.
Low-acuity urgent care	Response teams to assess and treat unscheduled, non-emergent needs such as general illness, minor wounds and injuries; mechanisms to initiate follow-up care in the community without involving the emergency department.
Mental health and situational crises	Crisis response teams – which may include a combination of paramedics, nurses, social workers or police officers – for clients experiencing a mental health or situational crisis. Clinical guidelines and criteria to directly refer or transport clients to designated mental health facilities.
Palliative care	24-h pain and symptom management provided by paramedics for clients rostered to a palliative care team. Provision of comfort measures to support clients’ wishes to remain at home rather than go to the hospital at end-of-life.
Public health and vaccines	Paramedics supporting public health initiatives by providing in-home and on-site vaccinations, distribution of naloxone kits and public education programs.
System navigation, case finding and needs assessment	Based on data on increased service use or observations at the scene of an emergency, paramedics initiate home visits to discover unmet needs and referrals to follow-up services, including connecting clients with primary care teams, case management and services that address social determinants (e.g., nutrition, income, transport).
Transitions to home after hospital discharge	Paramedics providing clients in-home assessments and wellness checks after discharge from hospital to identify unmet health and social needs. Paramedics serving as a ‘bridging’ service before home and community care supports are activated, and providing treatment for symptoms that can be managed in the home setting.

When asked about the rationale and underlying purpose of creating new programs, participants identified multiple overlapping drivers and conceptions of value. As illustrated in Table 3, program goals included improving client experience, reducing pressure on hospitals and emergency services and addressing needs for after-hours on-demand care. Programs also looked to fill specific gaps in local services and address inequities in care access. Success was defined as meeting multiple objectives rather than just one:

“Our definition of success is we should see an improvement in ED [emergency department] access. We should see an improvement in patients’ length of stay. We should see an improvement in ALC [alternate level of care]. We should hear from patients that they, from a patient experience perspective, are recognizing that their care is more integrated and they're not having to constantly repeat their story and their situation [ … ] from a fiscal management perspective, I should see a better return for investment of service versus a constant revolving door.” (Participant 10; Nursing, Occupational or Physical Therapist)

Participants identified a number of enablers and barriers, or factors that contributed to program success, detailed in Table 4. These factors included: influential leaders and managers; building trusting relationship networks between organizations, professionals and physicians; navigating challenges with shared data and information systems; and managing legal risk. These were reported as contributing factors rather than enablers and barriers because participants had variable experiences. For example, some participants described strong physician buy-in as an enabler, while others highlighted a lack of buy-in as a barrier. Similarly, some were successful in setting up shared medical records between paramedics and other agencies and identified this as a key enabler, while others were not successful and found lack of access to clinical records to be a barrier. In this way, participants described inconsistent experiences when implementing programs.

**Table 3:** Drivers, rationale and purpose of new models of care in Ontario

Driver, rationale or purpose	Illustrative Quotes
**Appropriateness of care pathway**: right type of care for a client’s needs; improving comfort and client experience	“Patient experience has been, is a very key factor in in seeing the benefit of these programs.” (Participant 19; Care Manager)
	“So how can we take the patients that are calling 911 anyway and help them get the treatment they need, get the relief that they need, and not put them in a position where they have to go to the hospital if that’s not what their goals of care are?” (Participant 8; Paramedic)
**Systemic and operational benefit:** reducing emergency service and emergency department use, addressing hospital overcrowding, cost reduction	“They also looked at 9-1-1 avoidance and things like that, which-- 9-1-1 calls did go down from this population when they knew this service was available. So that was kind of one of the metrics. They also looked at emergency department visits and things like that.” (Participant 1; Paramedic)
**Access to care:** increasing access or touchpoints to health and social services, particularly for hard-to-reach or under-served populations	“How do we narrow our focus? And that vulnerable population was kind of our threshold or lens to say, okay, yes, we can include you in our criteria” (Participant 6; Paramedic)
	“All I know is where our team specifically goes. And I think that this is kind of across the board in a lot of places. It’s where nobody else is going.” (Participant 14; Paramedic)
	“And what we were finding is that is how we were coming up with most of the issues and problems and discovering them, was those room-to-room wellness checks, because they were very apprehensive to come to us.” (Participant 16; Paramedic)
**Need for a mobile, timely, flexible 24/7 resource:** paramedics have mobility, staffing, equipment and logistics infrastructure to deliver a service on-demand	“So what is also key for them, for us, I find is their responsiveness to go in and do quick visits within 24 to 48 h.” (Participant 10; Nursing, Occupational or Physical Therapist)
	“So when a discharge coordinator is picking a service to refer a patient to, sometimes they pick paramedicine because they know it’s rapidly mobilized without question.” (Participant 18; Paramedic)
**Gap in service**: unmet local needs for reasons such as: resource shortages, clients not qualifying for services from other providers, jurisdictional issues	“So there’s people in the community, I think, that are lost in the system. So they’re getting missed because maybe they don’t qualify for homecare, but the hospital discharged them because we don’t have facility to keep them in beds at the hospital.” (Participant 3; Paramedic)
	“When we started to look at rolling out the CP [community paramedicine] program, we identified, as I said through the retrospective analysis, where are we seeing the pressures? And then from that, how do we engage with our community partners to say, how do we, you know, work to resolve this together?” (Participant 26; Paramedic)

**Table 4:** Factors that contributed to program implementation success or failure in Ontario

Contributing Factor	Illustrative Quotes
**Interprofessional trust and understanding**: Role negotiation and role sharing between different professions. Time spent building trust and overcoming territorialism.	“I think the first step is building these relationships, building the rapport, building the confidence and the trust. You know, I call it like an interprofessional trust and connection. Because without that, you can put the system in place, it’s not going to work.” (Participant 5; Paramedic)
	“I think we need to stop thinking of ourselves from the provider perspective and from what we do, and reverse that view from the patient perspective. And that’s where the integration comes in. It shouldn’t be paramedics do X and nursing does Y and physicians do Z and occupational therapy does whatever, and nobody talks to each other.” (Participant 6; Paramedic)
**Key role for physicians** in providing guidance, consultation, delegating medical acts, acting as a champion for programs amongst other physicians. Mechanisms to reimburse physicians for consultation.	“But I think it would only allow you to expand your own profession in a direction where physicians like myself aren’t, right? I don’t want to drive your profession, I want to work with you to drive your profession, right. And I think we need to build that.” (Participant 9; Physician)
	“Because we know that physicians listen to physicians better, you’re championing the program. You’re like, this is really worth it, we should do this for this reason, you’re trying to kind-of convince the other people and champion it in any way so that it’s more of a successful program.” (Participant 17; Physician)
**Organizational and personal networks:** Multiple organizations regularly meet and discuss issues. Paramedic representation at multiple “tables” – municipal, LHIN, hospital, OHT. Personal relationships between managers of local agencies.	“So now we’re involved in the OHT. But before that even came about there were, the LHIN had regional anchor tables, right. And so we were participants in an anchor table. And even before that the chief had brought together... public health made it one of their, I think it was for 2012, made it one of public health goals to look at sort of community paramedicine and brought a bunch of different disciplines to the table.” (Participant 25; Paramedic)
	“[Region] is unique in that regards, in that we kind of all know each other. So good and bad. But you do know, like, who all the players are, right? So I have a fairly close relationship with the manager for Home and Community Care for this area where we work.” (Participant 4; Paramedic)
**Buy-in from the frontline**: Providing career choice, selecting the “right” people for new roles, involving frontline providers in program design, positive or negative feedback that reinforces (or discourages) new practices.	“But, but kind of my takeaway from that is that you really need a willing and engaged workforce with high morale and feeling like they’re making a difference, and they’re seeing it, which is then kind of makes you want to do more, right?” (Participant 17; Physician)
	“I’m really grateful that we have those two individuals, because if you get the wrong person in that role, it could be very detrimental to the program.” (Participant 18; Paramedic)
**Information and data sharing:** Challenges with access to clinical viewers, navigating privacy law, siloed patient record systems, paramedics not legally seen as health providers.	“The biggest issue that’s out there with healthcare in general is the information sharing and privacy and the PHIPA, which is often misunderstood, right? And so there’s always the questions of whether or not that we can share information with our stakeholders and vice versa. And we all have different software platforms.” (Participant 13; Paramedic)
	“Privacy committee of [family health team] were adamant: nope, we’re concerned about opening up to other people to be in our health record. And this persona that you believe the record is yours, like your property. It’s the patient’s record. We’re all healthcare professionals.” (Participant 10; Nursing, Occupational or Physical Therapist)
	“And that, again, like we have these hurdles, we’re trying to get access to ConnectingOntario, but we’re not seen as either - we’re not a healthcare provider.” (Participant 15; Paramedic)
**Education, knowledge and decision-making:** Systems for paramedic education and learning, both entry-to-practice and ongoing; shortcomings in existing systems (e.g., staff hours, curriculum).	“So, for example, post secondary institutions to support training, development, and accreditation of these roles. We don’t have that in place. And we’ve seen that because we’re currently trying to figure out: how do we better align these professional development programs that we’re seeing throughout the province.” (Participant 26; Paramedic)
	“It’s a culture divide within paramedicine. So, older paramedics compared to younger paramedics, I find that education is lacking for the older senior paramedics, just in their initial education becoming a paramedic, there wasn’t a lot of focus on kind of substance use as well as mental health and kind of like, the whole biopsychosocial spectrum for that matter.” (Participant 14; Paramedic)
**Regulatory limitations of paramedic practice:** Prescribed scope-of-practice in law, requirements to transport to hospital-based services.	“And the problem - I think across Canada, more so in Ontario based on some of the laws - is that paramedics respond, as you’re aware, they have a choice to either transport to hospital or patients need to refuse care. There wasn’t any other sort of mechanism to make choices around that.” (Participant 9; Physician)
**Liability and risk:** Multiple actors – Ministry of Health, municipalities, individual physicians and paramedics – navigating concerns about liability and risk of adverse events.	“And I think part of the other issue was our medical director worked for them, is working for the region, right? And we’re working for the region. And then we have risk and legal over there who - and I think there were questions on does that - does she need more insurance? And who’s paying for that?” (Participant 15; Paramedic)
	“The other thing is, there’s this fear of risk and liability which is often, I think, misplaced, but it comes from the culture and the education. Right from day one when they start receiving education right through their career, it’s hammered into them that, you know, they have to cover themselves in case something goes wrong.” (Participant 13; Paramedic)
**Leadership and power:** Key leaders in influential positions that support or promote innovation.	“So from the community paramedic perspective, the best thing that ever happened to the group up here was they found the right person as leading the team that really gets it.” (Participant 10; Nursing, Occupational or Physical Therapist)
	“I cannot under emphasize the importance of having a leader like [name], that is innovative, forward thinking, supportive, and willing to think outside the box and support growth and innovation the way he does. That was probably, of all of this, the most critical piece. Because we could just as easily find ourselves with a leadership team that, you know, is very much by-the-book.” (Participant 25; Paramedic)
**Funding:** Inconsistent, transient, rolling funding envelopes; programs would have to shut down, re-staff, change shape; political funding priorities.	“We didn’t know, you know, we don’t find out until two or three weeks into the next fiscal year if we even have base funding. And that just seems to be an overall theme that, you know, no one really knows where the funding is coming from or what pocket of funding we’re going to be part of.” (Participant 1; Paramedic)
	“The biggest problem with it is that it was that short-term funding. So it would take us a couple months to get off the ground, build our clients, get the referrals basis, and then funding would end a very short time after that … probably one of the worst things was that after you get it up and running, you lose the funding and then you completely start over at ground zero the next, next pilot. You have to try and rebuild all those networks and build those referral pathways and things like that.” (Participant 16; Paramedic)

### Experiences with implementing models of care

Thematic analysis of participants’ experiences in implementing these models of care – including the drivers, enablers and barriers described above – resulted in three main themes and a number of sub-themes, summarized in Table 5 at the end of this section. These themes are:

1. Adapting and being nimble in tension with system structures
2. Evolving and flexible professional role identity
3. Unpredictable influences on program implementation

#### Adapting and being nimble in tension with system structures

Participants described navigating tensions between nimbly responding to local needs and inflexible system-level structures, including regulation, standards and funding. These tensions manifested in different ways, reported along two sub-themes: (1.1) local and distributed versus standardized and centralized control; (1.2) historical mistrust and “working around” regulatory barriers.

##### Local and distributed versus standardized and centralized control

Many programs were developed and led by networks of local actors, including municipal paramedic services, primary care teams, home care and community agencies. While some participants valued having local control over the scope of their programs, others highlighted the need for some standardization across the province but questioned to what degree that was possible without losing ownership and “local flair” (Participant 21; Nursing, Occupational or Physical Therapist).

“There's a lot of local variation in local policy and players and actors involved that make it very difficult to standardize an approach - however evidence-based it might be - for different regions.” (Participant 13; Paramedic)

“There's too much that can fall apart and, and I think that there needs to be some consistency and standardization for programs across Ontario.” (Participant 18; Paramedic)

The tensions between local needs and standardization were also discussed in relation to program funding. Participants described challenges with seeking sustainable funding due to a lack of provincial recognition. This led to some programs being temporarily funded by municipal governments that saw value for local constituents; however, municipalities were reluctant to permanently fund health services, which are a provincial responsibility.

“We're not standard. We're not - we don't talk the same language, we're not defined by the same policy procedure, we don't have the same scope, we don't have the same competency. And that's become a challenge when you try to fund something.” (Participant 5; Paramedic)

“I went to council and presented and pleaded for money. [ … ] [They recognized that] we can't let it end at this point of time so they agreed to bridge until the end of 2020 while we looked for other funding agents.” (Participant 18; Paramedic)

Participants identified that existing quality assurance and clinical oversight mechanisms are inadequate, but disagreed on solutions. Some suggested that the centralized, standardized approach of the Base Hospital – the clinical regulatory body for paramedics – is fundamentally incompatible with locally-driven models of care; others advocated for more Base Hospital involvement to address gaps in oversight. Participants highlighted that the Base Hospital process to define medical scope for paramedics (i.e., aiming for province-wide protocols, developed by a small committee) does not allow local variation, nor does it allow sufficient inclusion of non-emergency physicians, allied health and paramedics themselves in collaborative decision-making.

“It's like always sticking a square peg into a round hole because the one size doesn't fit all.” (Participant 11; Paramedic)

“I think that is a really important positive piece we're getting from this, is the collaboration from different health groups and the perspectives and you know, that insight, and all of those different voices to be able to contribute to put together the best plan for the patient. Versus having one group dictate everything.” (Participant 6; Paramedic)

In the absence of mechanisms for local control and clinical oversight, programs innovated by obtaining medical authorization from local physicians, including family physicians, internal medicine and medical officers of health. This led to inconsistency in paramedic education, quality assurance and documentation processes, making it difficult to measure quality or perform program evaluations.

“It's a bit of a wild west of delegation, it ultimately then comes down to you how do you do QA [quality assurance]? How do you do remediation? How do you do all of those things which are part of the day-to-day practice of what a base hospital does. But in community paramedicine there's no framework for that, at all.” (Participant 24; Physician)

Participants highlighted that the Base Hospital system is now investing in systems and processes to enable future models of care in the province, but early experiences with this are mixed. Some local areas participating in these new initiatives feel held back because of persistent delays due to the Base Hospital’s drive for regional standardization.

“And I had to shake my head and then I was stalled. I was stalled numerous times to the point where it's two years later [ … ] And I'm still waiting for the rollout across the entire region, base hospital region that is, where some of those services aren't making it a priority.” (Participant 11; Paramedic)

##### Historical mistrust and “working around” regulatory barriers

Participants described a reluctance to engage with the Ministry of Health due to ongoing and historical experiences with innovation.

“I think the negativity around Ministry laws, Ambulance Act, I think community paramedics are trying to stay away from because to change things and to be nimble has never been the positives of the Ministry [ … ] if we want to add a directive or a new care model, it can take four years, right, because it's not nimble, it doesn't change. Whereas right now, with the community paramedic world, things are changing daily.” (Participant 9; Physician)

Multiple participants told stories of programs that had failed because when they chose to seek approval, they were prohibited from proceeding. They saw regulatory bodies as “very, very slow,” “risk averse” (Participant 13; Paramedic), unwilling to experiment and uncomfortable with ambiguity.

“But the biggest barrier was that we ultimately never got Ministry of Health approval [ … ] there was a bit of gray area when we would have our medical directives, which was not welcomed by the Ministry.” (Participant 15; Paramedic)

Despite recent regulatory changes that enable some new models of paramedic care [17], participants suggested the new frameworks continue to be prescriptive and do not allow enough flexibility.

“So right now what's happening is the Ministry is really focusing on limiting the number of these alternate patient care models that can happen. They want to have, you know, one going and going well before they look at another one.” (Participant 2; Physician)

Participants used creative strategies and work-arounds to legal and regulatory barriers when implementing programs. For example, participants described how labeling certain roles and programs as ‘community paramedicine’ rather than ‘paramedicine’ has been a key strategy in avoiding legal “roadblocks” (Participant 2; Physician) to what paramedics are permitted to do.

“Our legislative body is the Ambulance Act and Reg 257. Within that, it defines what a paramedic is. It does not define what a community paramedic is. And because of that, community paramedicine does not fall under the Ambulance Act, and has that, and as a result, we have that latitude, that flexibility.” (Participant 26; Paramedic)

Some participants specifically described strategies to navigate limitations such as paramedics only being allowed to transport clients to hospital-based facilities and lack of shared medical documentation between paramedics and other providers.

“And we had to get really creative on how do we actually get to even pilot this to, you know, to get off site? You know, can we designate a room within [mental health organization]? Do we have to designate [mental health organization] under [hospital]?” (Participant 26; Paramedic)

“We created these stickers. Because so, legal, legal issue. We're not allowed to document in the chart in the home binders that exist in every palliative care person's home.” (Participant 8; Paramedic)

Participants acknowledged that some workarounds are “not efficient” but that they do what “needs to be done” (Participant 23; Physician). When asked what legal or regulatory changes they would like to see, some found it difficult to specify because they are so accustomed to working around barriers that they don’t necessarily see them as barriers any more.

“We're used to just navigating around so many barriers that I don't even know what's really necessary anymore and what we can kind of cut and paste together to get done what needs to be done.” (Participant 14; Paramedic)

#### Evolving and flexible professional role identity

Participants highlighted the ongoing evolution of the paramedic profession and flexibility in role definition. Sub-themes included: (2.1) key leaders with conviction for change, (2.2) role flexibility as a core value, and (2.3) divergent views in the workforce.

##### Key leaders with conviction for change

Senior management in some paramedic organizations were seen as central to driving new programs. These leaders actively searched for new roles for paramedics to play; they had a “vision” (Participant 15; Paramedic) and looked to increase the profile of paramedics in the system. At local meetings and committees, these leaders volunteered to fill gaps in services.

“So [meeting of local agencies] kind of came together and said, "who could help out?" And my deputy chief is always looking to kind of expand the role of paramedics and [they] put up [their] hand and said, "oh, we can help out for sure.” [ … ] A lot of our success is tied to the leadership that we've had and kind of this, you know, this interest to be integrated and recognized as a healthcare provider in our area.” (Participant 1; Paramedic)

The leaders also had conviction to sustain new programs and initiatives despite challenges such as lack of funding and regulatory barriers. Once programs were established, they actively advocated for their continuation with policymakers and decision-makers.

“We were promised funding, but we didn't actually see it for a little while there. But, you know, luckily our CAO [manager] was immensely supportive of this. And [they] said, "You know what, it's the right thing. Just do it, we'll figure out the funding later."” (Participant 7; Paramedic)

This conviction for expanded paramedic roles was supported by a grassroots network across the province. Through this network, paramedic leaders shared tips, documents and resources to implementing programs and navigating challenges.

“So we had kind of already developed these community of practices to share, you know, "how are you doing this?" and "we've got a last minute request to swab 1700 people, what have you done when that request came to you?" So there was a lot of kind of conversation and dialogue happening.” (Participant 6; Paramedic)

##### Role flexibility as a core value

Participants described an underlying action-oriented culture in paramedicine as a core part of the profession’s value-add to integrated care. Some described that working in uncontrolled emergency settings means the profession has a generalist skillset, comfort with ambiguity and a willingness to “gap fill” and “do what it takes” (Participant 22; Paramedic). Participants resisted specifying the role of a paramedic, and referred to paramedicine as a “mobile enhancer” (Participant 11; Paramedic) to the system that morphs depending on local needs.

"So I think we have different training than nurses, we have a different training than physicians [ … ] we're more generalists and we're more, you know, the culture of a paramedic is: let's get this shit done. And let's, let's fix the problem. You know, like you're on the road and, you know, you arrive at a situation, whatever the situation is, you dig away, you know, how can we fix this?" (Participant 5; Paramedic)

Participants also suggested that the trust the public puts in paramedics – including the image of wearing a uniform – contributed to some programs’ success. However, others identified misconceptions of the paramedic role amongst allied health and the public as a challenge to be overcome with messaging and education.

“Also, the community has a little bit of a misconception on what paramedics are and what they do. A lot of people still see paramedics as ambulance drivers [ … ] So it took a lot of presenting and took a lot of just networking to open up people's eyes to what paramedics can actually do and the range of our scope and how we can even evolve into other areas and aspects of community care.” (Participant 16; Paramedic)

Participants described how a flexible and variable role definition contributed to interprofessional tensions. They expended considerable efforts building trust with partner agencies and allied health, and assuaging concerns that paramedics were taking away jobs from other professions such as nursing. Most succeeded in establishing productive relationships over time.

“So like the biggest thing has been connection and conversation and developing those relationships with your partners and assuring them that we're not there to scope-creep. We don't want to do their job. We're there to help them, we're there to support them and do whatever is right for their patient. And I think that that messaging has been incredibly important to make this pilot successful.” (Participant 8; Paramedic)

“Getting your partners to understand you can trust us, we'll get it done. [ … ] My superintendents and myself spent a lot of time - we called it our traveling roadshow - meeting with [region] LHIN home care coordinators, meeting with other healthcare system partners. Here's what our program's about, here's what we can do. We want to do more, we can take on more, like essentially, it was like a propaganda tour” (Participant 22; Paramedic)

##### Divergent views in the workforce

Participants described a range of willingness versus reluctance in the paramedic workforce to embrace new roles, and some suggested this was a generational shift in the profession.

"So you're gonna have some paramedics who are very opposed to change. And I think that's probably more of the older ones who don't want to have this, you know, medical decision process going on. You're gonna have the middle ones who are going to be okay with it, maybe not embracing it completely. And then you're going to have some that are going to embrace it completely, like they want to see this change happen. And it's going to be-- they understand it's going to be the future of paramedicine." (Participant 2; Physician)

New roles were also suggested to improve job satisfaction and engagement. Participants indicated that more career options in paramedicine can contribute to provider wellness, including alternate roles when paramedics are physically injured.

"You know, paramedics for years and years and years have gotten into the job, loved it and then went, okay, now where do I go from here? Because there weren't- there weren't a lot of different, you know, secondments or career options within the industry or within the service. So the fact that there are now, they seem very excited about." (Participant 22; Paramedic)

Ultimately, participants stated that where possible, providing providers with choice – as to whether they participate in new roles – was one strategy in finding the right types of people for new roles and functions. Providers being “part of the conversation” (Participant 11; Paramedic) when developing new models was identified as key to success.

"And we've never forced anybody to go to the [program name]. It's completely voluntary. So the paramedics that do go there really believe in the program and want to be there and want to help support." (Participant 7; Paramedic)

#### Unpredictable influences on program implementation

In implementing new programs, participants highlighted the unpredictable influence of: (3.1) the COVID-19 pandemic; and (3.2) political priorities.

##### COVID-19 as an accelerant to pre-existing trends

Most participants described how the COVID-19 pandemic rapidly accelerated the implementation of new models of care that were previously facing barriers. Participants described how health system pressures resulted in a willingness to try new things across the system. The pandemic also enabled access to funding and breaking through bureaucratic hurdles.

“It [COVID-19] has strained the system, but it's also been a good opportunity for growth. Whereas before, you would submit that - I don't know, I know we tried to get flu shots here for the longest time for our paramedics to deliver flu shots and it was like, our application was turned down a couple years in a row. And then all of a sudden, this year, it's like stamp stamp approval approval.” (Participant 18; Paramedic)

“There's a phrase that we have been using a lot, it's "never waste a good crisis." And with COVID, we got a lot of funding.” (Participant 8; Paramedic)

COVID-19 was also credited with putting a spotlight on frontline workers. This helped challenge misconceptions and allowed paramedics to demonstrate their value-add amongst the public and allied health agencies.

“The last year has been transformative for Paramedic Services directly as a result of the pandemic, like it has been for the entire healthcare system … one of the enduring positive legacies, I think, is that the profile of the paramedicine profession and what services and the practice -- paramedic practitioners can provide” (Participant 22; Paramedic)

While COVID-19 was an accelerant, pre-existing relationships and collaborative infrastructure were key to allowing programs to adapt and play new roles. This included the existence of local working groups, committees and Ontario Health Teams.

Ontario Health Team was created before COVID hit - so they [paramedics] already had a seat at the table. And so they're there for every meeting. And so they were included on all the thought, thinking processes of anything, and they put their hands in everything. (Participant 17; Physician)

##### Changing political priorities

Participants described unpredictability due to politics and political priorities; for example, suddenly being able to implement a stalled program “because the government changed” (Participant 14; Paramedic). They also described a randomness in funding, including being denied funding for locally-developed proposals only to then be provided funding for a government-driven province-wide initiative.

“It was the ministry that came out and offered it. So they said they were starting community paramedicine across Ontario, we are one of the services that signed up for it, didn't really know what we were getting into at the time.” (Participant 16; Paramedic)

“A group of us in [region] got together and we wrote three funding proposals specific to community paramedicine … And none of those proposals received a response. But we recently were given two other funding models that we had not applied for.” (Participant 6; Paramedic)

Participants expressed a frustration with the role of politics in healthcare. One participant described delayed approval of a program followed by a pressure to implement on an unrealistic timeline, driven by government intention to make a public announcement. There was a general sense that “there is way too much politics in healthcare” (Participant 10; Nursing, Occupational or Physical Therapist) and healthcare priorities should be set by professionals rather than politicians.

“We initially put our requests to the province in as part of the patient model standard in September. We didn't hear about it till February. And then they said, you need to tell us when you can actually have this happening for patients on the road. And it needs to happen in 2021 because the minister wants to announce it. Like it was all about the PR piece. And we're like, we're in the middle of a bloody pandemic. And, you know what, we'll let you know, probably won't happen till early 2022.” (Participant 22; Paramedic)

**Table 5:** Themes that represent participants’ experiences implementing programs and models of care in Ontario

Theme	Sub-theme	Summary of Concepts
1. Adapting and being nimble in tension with system structures	1.1. Local and distributed versus standardized and centralized control	- Programs developed collaboratively through local networks of providers and organizations to meet local needs, value local control. - Lack of standardization created challenges for quality assurance and funding. - Existing centralized systems for clinical oversight were inadequate, with no mechanisms to share power with local providers.
	1.2. Historical mistrust and “working around” regulatory barriers	- Ministry of Health and regulatory bodies seen as slow and risk-averse, history of denying program approval. - Creative strategies used by programs to avoid and work around regulatory and legal barriers.
2. Evolving and flexible professional role identity	2.1. Key leaders with a conviction for change	- Some leaders within the paramedic profession actively pushed for new roles, eagerly volunteering to fill local service needs and advocating for program funding. - Conviction to program implementation despite challenges such as regulatory barriers and lack of funding.
	2.2. Role flexibility as a core value	- Paramedics seen as flexible, mobile gap-fillers in local systems. - Reticence to define paramedic role; ambiguity and flexibility seen as a value-add. - Lack of role definition contributed to interprofessional tensions.
	2.3. Divergent views in the workforce	- Generational change within the paramedic profession – some eager and some reluctant for new roles. - New roles contributed to job satisfaction and desirable career pathways.
3. Unpredictable influences on program implementation	3.1. COVID-19 as an accelerant to pre-existing trends	- Health system pressures and urgency due to COVID-19 enabled access to funding and overcoming of bureaucratic hurdles that previously existed. - Paramedics were in the spotlight, leading to recognition of potential value-add of new roles and functions.
	3.2. Changing political priorities	- Unpredictable approvals for programs due to changing government focus. - Disconnect between local needs and political funding priorities. - Frustration with the role of politics and politicians in healthcare.

## Discussion

This study found that collaborative networks of organizations in communities across Ontario created diverse programs in response to local needs, which is a desirable characteristic in integrated care [1, 2]; the results offer insight into governing health systems towards being more integrated, adaptive and responsive. The programs’ responsiveness to population needs were driven by strong local relationships and an eagerness amongst the paramedic profession, which was willing to defy traditional role definitions in health and social care. This suggests that empowered local leadership and availability of a workforce that is flexible, mobile and willing to change roles may enable integrated care and increase a system’s ability to adapt [29, 30]. However, programs navigated tensions and developed work-arounds to legal and regulatory systems that were built for central control and standardization, which did not allow for sufficient local control or practice variation. While employing work-arounds allowed programs to be nimble and responsive, it created challenges with consistency, quality assurance, performance management, data sharing, funding and interprofessional role delineation. These findings can be discussed in terms of the degree to which the governance structures of a health system enable or inhibit integrated care. The results of this study add to the discourse on applying the principles of network governance and complexity thinking to health system governance in two ways: (1) setting system rules and structures; and (2) experimentation versus institutionalization. Each of these are discussed below, followed by some implications for practice.

### Setting system rules and structures

A key role of health system governance is to create the social and structural conditions for integration and adaptation to emerge amongst networks of actors throughout the system [2, 6]. This is premised on the idea that the overall behaviour of a health system is not completely predictable, controllable or planned, but rather emerges from the dynamic interactions between clients, professionals, organizations and events [2, 6, 21]. A network governance approach involves influencing the rules by which actors interact – for example, through flexible frameworks, incentives, shared vision, messaging and organizational structures – and thus impacting the overall system’s behaviour [6, 21]. This study found that the mandated existence of certain network-based structures in Ontario – the LHIN’s and more recently OHT’s – contributed to local adaptability by facilitating relationships and trust amongst local organizations. These networks were able to problem-solve and mobilize resources to address local needs; this is encouraging, and is supported by the literature on organizational contexts that enable integrated care [31]. However, these networks appeared to be loosely-governed [7], relying primarily on good-faith relationships between specific individuals often without formal administrative bodies. There appeared to be an absence of province-wide shared frameworks, vision or messaging, for example regarding the goals and areas of focus for new programs, the role of paramedics, regulatory oversight and performance management. Instead, individuals and groups – including physicians, care coordinators and some paramedic managers – chose to develop and implement programs of their own accord, driven by their own motivations, without a coherent set of rules and frameworks. This may point to missing elements in overall system governance – e.g., shared vision, frameworks and supporting regulation – resulting in potentially fractured, unintentional and unfocussed programs across the system. This network behaviour may – to a degree – be desirable, as it allowed local leaders to act on local priorities; however, the challenges they encountered suggest that the rules and structures of the system did not intentionally support or encourage them to do so. A lack of supporting regulation, shared vision and quality frameworks are not desirable, and can decrease the effectiveness of integrated care programs [31].

### Experimentation versus institutionalization

The findings of this study also highlighted the tension between experimentation and institutionalization in enabling integrated models of care. It has been suggested that governing health systems towards being more adaptive and responsive to population needs might involve striking a balance between the system’s “complexity leaders” and its “formal (traditional)” leaders [6]. Complexity leaders operate in a system’s “experimental arm,” and are given the time and space to innovate to continually solve emerging system problems. Meanwhile, formal leaders in the system’s “institutional arm” – in addition to facilitating networks and setting system rules – institutionalize successful innovations using more traditional, top-down strategies [6]. The programs in this study together represent an experimental arm in Ontario’s health system landscape, lead by a distributed network of complexity leaders in communities across Ontario. In implementing these locally-driven programs, however, complexity leaders encountered certain inflexible system structures – e.g., regulations, clinical oversight – that did not allow for innovation, and were built for centralized rather than distributed leadership. In the absence of appropriate space to be nimble and experiment, the complexity leaders employed strategies to avoid and work around system structures. This behaviour was also informed by a skepticism of the system’s institutional arm – the Ministry of Health and the Base Hospital. The institutional arm was sometimes seen as politically-motivated, slow and risk-averse, and complexity leaders were skeptical of the institutional arm’s ability to support innovation. Furthermore, the institutional arm’s recent approaches to ‘scaling up’ some programs have been to standardize activities, continue to use centralized clinical oversight and limit the types of models of care that are allowed [17] – in some cases this had led to province-wide roll-outs of programs that are not necessarily appropriate for all jurisdictions. While institutionalizing and standardizing successful innovations is one role of governance [6], it is equally important to maintain space for ongoing experimentation, local leadership and local flexibility. In the absence of this, the system may fail to take full advantage of the adaptive, responsive problem-solving power in its experimental arm, as evidenced in this study. Furthermore, maintaining an overly restrictive, standardized approach risks reinforcing the historical skepticism that is now built into the system. This may perpetuate an unwanted and inefficient pattern of behaviour, where complexity leaders innovate in a vacuum and traditional leaders fail to take advantage of it to achieve system-level integrated care goals.

### Implications for health system governance

The findings of this study point to some health system governance strategies to increase adaptability and responsiveness in integrated care systems. These strategies include:

- support local relationship networks around a shared vision or framework;
- build trust with local leaders through systems that allow sufficient local control;
- consider the value of a functionally flexible health workforce;
- develop guidance, legal and regulatory allowances for ongoing local experimentation;
- choose strategically when to standardize programs; and
- tolerate some inconsistency across the system

These findings support and add to the existing integrated care literature on network governance structures [7, 12], flexible workforce and staffing [32], the importance of shared mental models [8] and taking a tolerant, hands-off approach to system governance informed by complexity thinking [2, 6]. The findings also provide further evidence against top-down, command-and-control policy programmes that continue to dominate the landscape of integrated care efforts [2, 33–35]. Finally, the grassroots emergence of new models of paramedic care suggests that valuable roles and functions of different professions can be discovered by systematically including a broad set of providers and organizations in local integrated care relationship networks. This study provides insight into the potential role of paramedics within integrated care, which may be to fill the unmet need for flexible, mobile, re-deployable staff in local systems. Further work is needed, however, on understanding the paramedic community’s interest, suitability and specific value-add in the skill mix of integrated care teams and building appropriate system structures to enable these functions.

### Limitations

Shortcomings of this study include: (a) missing perspectives; and (b) recency bias due to the COVID-19 pandemic. The purposive and snowball sampling strategies used in this study means that the findings disproportionately reflect the perspectives of people who championed new models of care, and thus excluded communities and organizations that chose not to implement new programs or failed to get them off the ground. This may mean that our understanding of barriers to implementation is incomplete, and that our results over-represent the positive aspects of new models of care. While some findings indicated how frontline staff responded to programs, the perspectives of average paramedics, nurses and other providers were not sufficiently captured; however, our findings on divergent views amongst the paramedic workforce are supported by previous studies [36]. None of our participants had worked directly for the Ministry of Health and as such the government perspective is missing, so our results likely depict an incomplete view of regulatory challenges. Equally important, patients and their caregivers were not part of our sample and their experiences with these programs are an important area of future study. Secondly, this study was designed prior to the COVID-19 pandemic, but data was collected while Ontario was experiencing significant second and third waves of COVID-19 infections. The pandemic shifted the roles of providers in the Ontario health system and resulted in rapid changes in practice; this likely impacted what participants chose to speak about due to recency bias. We tried to account for this by asking participants to describe historical experiences with programs, but nonetheless recent changes in participants’ perspectives due to COVID-19 potentially influenced how they recalled past events, and thus impacted our results.

## Conclusions

This study provided insight into the value of organizational networks, local leadership and a flexible, generalist workforce as necessary components of an integrated, adaptive and responsive healthcare system. The findings also highlighted some complex challenges for health system management in integrated care, such as: enabling appropriate levels of experimentation in the system, building trust between complexity leaders and formal leaders, and balancing standardization with flexibility. Further work is needed to develop guidance for policymakers and managers to strike a balance between these tensions, and manage health systems towards being more integrated, adaptive and responsive.

## Data Availability

The datasets generated and/or analysed during the current study are not publicly available due to them containing information that could compromise participant privacy, but are available from the corresponding author on reasonable request.

## Abbreviations

CP: Community Paramedic (ine)
COVID-19: Coronavirus Disease 2019
LHIN: Local Health Integration Network
LTC: Long-Term Care facility
OHT: Ontario Health Team
